# A Step Towards Rational Diet

**Published:** 1910-09-15

**Authors:** C. Lucey Palmer

**Affiliations:** Newmarket


### A STEP TOWARDS RATIONAL DIET.

BY C. LUCEY PALMER (NEWMARKET) L.D.S., ENG.

In spite of the many advances which have been made in dental science, we dentists do really very little in the way of preventing our patients suffering from dental caries and irregularities, and it seems to me that there is a grand work for some influential body to do; and why should not the British Dental Association make an effort in that direction?

In the medical profession there is what is known as "preventive medicine," which has for its object the formation of a physically fit race of human beings, and probably it should be one of the functions of preventive medicine to investigate the subject of dental degeneration, but I fear it either does not do so, or the members of the medical profession are singularly slow in accepting its teachings as it seems to me that medical men are as lax as the laity as to their diet. So if a body of dentists can educate the public only a little as to what they should eat, and should not eat, they can claim to have been instrumental in the formation of what may be called preventive dentistry.

The medical inspection of school children has drawn attention, *inter alia*, to the dreadful condition of children's teeth, and much has been written about the treatment of these teeth, but can nothing be done to prevent future children getting into the same deplorable condition. I am strongly of the opinion that a great deal can be done, not only towards an improvement in the condition of their teeth, but also towards an improvement in the state of their alimentation generally, as I take it that carious teeth must be considered a sign of deranged health.

We must first consider to what cause we may attribute this abnormal amount of decay. Shall we say it is due to imperfect development of the teeth? or to deterioration after development has taken place I am inclined to think the primary cause is developmental, and to a great extent pre-natal, although the trouble is largely augmented by a continuance of the errors of diet in later life. To my mind, it is of the utmost importance to let our consideration of this matter begin with the early days of pregnancy, not only with regard to the teeth of the child, but also with regard to the health of the mother, for I am convinced that if that ever present constipation could be overcome we should not so frequently hear the statement made that mothers cannot nurse their children, either because they have no milk, or that milk disagrees with the children. If women will only keep themselves healthy with plenty of exercise, natural food, and no late hours during pregnancy, I believe the majority of them could nurse their children, in spite of what may be said by the medical and nursing professions. Given a healthy mother, living on plain, natural food, who can and will nurse her child, any such child must surely have a better chance in life than a child born of a mother who pays no regard to her health, and which is later fed by artificial means. The alternative to mothers milk is generally cows milk in some form or other, and I should like to call attention to the fact that the human being is an omnivorous creature, whilst the cow is a herbivorous ruminant, producing milk eminently suited to the rearing of other herbivorous creatures, but possibly not quite suited to the human race.

The fact of teeth being so bad is only a sympton of the fact that our diet is sadly amiss somewhere, and it is with regard to one very evident error that I wish to speak today, although there are probably several factors to be considered when speaking of dental caries.

Seeing that the last two generations have shown such a rapid degeneration as we are accustomed to see, and that dental troubles are pretty common to all classes, it is evident that the mischief is one which has had cause of origin in some article of diet which is generally consumed and comparatively recently introduced. Roller mills from being rare sixty years ago, have become gradually more common until now the bulk of our flour is produced by them, and with their introduction biscuit factories have sprung up. We are probably all agreed that the highly refined flour which is produced by these mills is the chief cause of dental degeneration, *but the public does not know it.* The object of this paper is to see if some suggestion can be made to draw public attention to this matter, for if we are agreed that it is a matter of importance it is surely our duty to enlighten the public.

If I am right in assuming that the cause of dental trouble is developmental, it is hopeless for the majority of the present inhabitants of this earth to expect to get any material improvement in their teeth, however conscientiously they eat nothing but wholemeal bread, but I firmly believe that a vast improvement will be noticed in the next generation in all families where white bread and biscuits are avoided and wholemeal bread takes their places.

There is probably not one in 100,000 of the public who knows that the calcification of the teeth is largly completed *in utero*, and that where calcification has taken place only very slight improvement takes place afterwards. The public does not consider teeth until it sees them or until they ache.

To begin with, the future mother must entirely abstain from eating bread and biscuits made from refined flour and replace these with wholemeal bread; this is to help to avoid constipation, in addition to providing the child with its natural requirements. During lactation the same care must be taken by the mother, and from the day that the child begins to eat anything until above the age of 16 this care must be maintained.

In my own practice I am continually lecturing my patients on this subject, but, alas! I fear with little success. One reason for my failures is that the public absolutely cannot purchase wholemeal bread, and that is because the millers will not produce wholemeal flour, except in very small quantities, and the bakers do not actually know how to make the bread, but will insist upon mixing some questionably wholemeal flour with their white meal sponge, and the resulting loaf is as dry as sawdust, whereas a loaf made from coarsely ground stone-milled flour is at its best from two to five days' old. What scientific knowledge has the miller or the baker of the physiological requirements of the human race with regard to this important article of diet? The processes of evolution have done much to enable the human race to adapt itself to a diet very different to that on which it originally subsisted, but surely the millers and the bakers are taxing these processes beyond their endurance in asking them to enable the human race to forego its wholemeal bread, on which it has subsided for many centuries, and to accept what they consider best for it; and all this in two generations?

I do not intend to go into the analysis of wholemeal flour; but suffice it to say, that it contains nothing but what we require to build up bone, tooth, and brain substances, and of most importance from a general, as distinct from a dental point of view, it contains, in its cellulose, a natural aperient whereas refined flour consists almost exclusively of starch and, as you all know, this is converted into cane sugar, then into grape sugar by the action of the ptyalin of our saliva, and the pancreatic juices; and as grape sugar it is absorbed through the intestinal mucous membrane to build up into fat; thus, we are not only deprived of our chief source of supply of bone, tooth, and brain-forming substances, but of our natural aperient, and to make up for the latter we fly to the patient-medicine vendor, who flourishes exceedingly.

The indegistible portion of the grain of wheat causes a slight irritation of the intestinal mucuous membrane, causing increased peristalsis, whereby not only is the intestinal content forced onwards but the very use of the muscles stimulates them to further development. This is infinitely better than that drugs should be taken which usually stimulates the digestive glands and have a purgative action.

I am very fond of pointing out to my patients that of all animals the human race suffers most, not only from dental caries but also all intestinal troubles, and that is because dumb animals are still living upon the same food as they did at the time they were created, and that the human race has altered its diet not so much by addition as by subtraction from its chief article.

For the proper developement of our teeth, I maintain that it is necessary for us to eat wholemeal bread up to the age of 16, and for the sake of our general health we must continue to eat it to the entire exclusion of white bread to the end of our days.

I have only incidentally mentioned biscuits, but in infancy they are even more injurious than white bread, because children are continually munching them both in bed and out of bed, and their teeth have continuously a coating of starch upon them and this after its conversion into sugar, is changed into lactic acid, which of course, has a direct decalcifying action on the teeth. Biscuits must be put in the same class as sweets.

There is another subject which has a great deal to do with perverted digestions primarily, and dental caries secondly, and that is with regarded to the consistency of food which is given to children to supplement, and later to replace, lactation, but as that is beyond the scope of this paper I will only refer shortly to it. It seems to be a general belief that children must have all soft food, such as bread and milk, minced meat and so on, but from the time that the first temporary teeth are cut surely this must be entirely wrong, as it tends to inculcate the habit of bolting food, which habit is never got rid of. Instead of the meat being minced or finely cut up, it would be infinitely better if children were given a piece too large to swallow, which would need masticatioa but I suppose asthetic objections would be raised to such n course being adopted.

Now we must briefly consider how it is that these errors have come about. I believe the origin has been in a lack of knowledge of physiological principles. Analyses of certain foodstuffs show that they are made up of certain substances to which the physiologist attaches certain values and the medical practitioners, forgetting the possibility of the innutritious substances having any value, have recommended foods in which the innutritious substances been largely eliminated demand has created supply, and now we have concentrated foods of all sorts, which are much vaunted by their vendors for commercial reasons. Unfortunately, mothers gain a lot of their knowledge about the bringing up of children from maternity nurses, who as a class are wonderful experts on the values of patent foods and who dearly love their biscuits and white bread. The elementary principles of physiology should be understood by all who have to do with children, and if attention were paid to these principles instead of to the analyses of patent foods, we should find healthier children.

In an exhibitions of bakers and millers produce, it always seems to me to be a great anomaly for prizes and diplomas to be granted for greatest purity and whitness of flour, yet this is an ordinary occurrance, and great use is made of these prizes and diplomas to advertise nothing but starch and fool the people. Government inspection should be extended to bread foods, so that people could know the real food values of what they believe is the staff of life.—*British Dental Journal.*

